# Diffuse Alveolar Haemorrhage as Initial Presentation of Systemic Lupus Erythematosus: A Case Report

**DOI:** 10.31729/jnma.3903

**Published:** 2018-12-31

**Authors:** Ashesh Dhungana, Prajowl Shrestha, Bhakta Dev Shrestha, Anil Baral, Gita Sayami

**Affiliations:** 1 Department of Medicine, National Academy of Medical Sciences, Kantipath, Kathmandu, Nepal; 2 Department of Nephrology, National Academy of Medical Sciences, Kantipath, Kathmandu, Nepal; 3 Department of Pathology, TU Teaching Hospital, Kathmandu, Nepal

**Keywords:** *Bronchoalveolar lavage*, *Case report*, *Diffuse alveolar hemorrhage*, *Systemic lupus erythematosus*

## Abstract

Diffuse alveolar hemorrhage results from accumulation of red blood cells in the alveolar space originating from alveolar capillaries. Alveolar hemorrhage in Systemic Lupus Erythematosus is rare but catastrophic and can rapidly progress to respiratory failure. We report a 22-year old lady who presented with dyspnoea on exertion, hemoptysis, bilateral leg swelling and oliguria. Diffuse alveolar hemorrhage was confirmed by bronchoalveolar lavage fluid analysis. Serologic tests and renal biopsy confirmed lupus nephritis. She was treated with systemic immunosuppressive therapy and plasma exchange, to which she had a favourable response. Lupus presenting as alveolar hemorrhage is rare which warrants prompt diagnosis and treatment to prevent complications.

## INTRODUCTION

Diffuse alveolar haemorrhage (DAH) results from accumulation of red blood cells originating from alveolar capillaries into the alveolar space.^1,2^ Systemic vasculitis and connective tissue disorders are common causes of DAH, where capillaritis in pulmonary microcirculation is the characteristic feature.^3,4^ Drugs, coagulopathy, infections and anti-GBM antibody syndrome can cause alveolar hemorrhage in the absence of pulmonary capillaritis.

DAH is a rare catastrophic complication in SLE which can rapidly progress to respiratory failure.^5^ We hereby report a 22-year old lady who presented with DAH which was diagnosed by Bronchoalveolar Lavage (BAL). Serological tests indicated presence of SLE and kidney biopsy confirmed presence of lupus nephritis. This case report highlights the importance of bronchoscopy and BAL in the diagnosis of DAH in appropriate clinical setting.

## CASE REPORT

**Figure 1.**
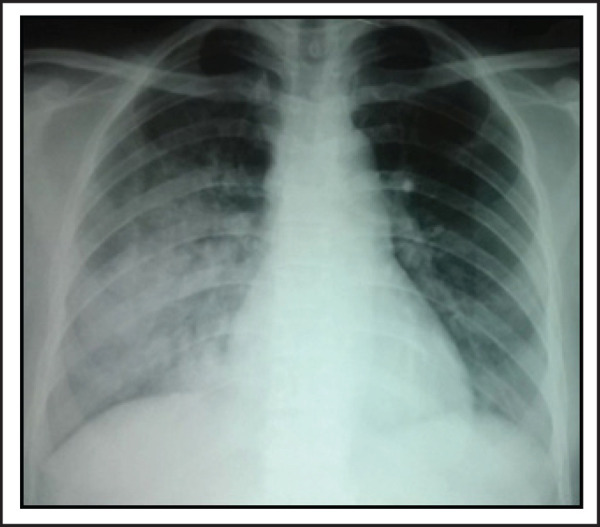
Chest X-ray showing bilateral alveolar opacities in the lower and middle zones.

**Figure 2.**
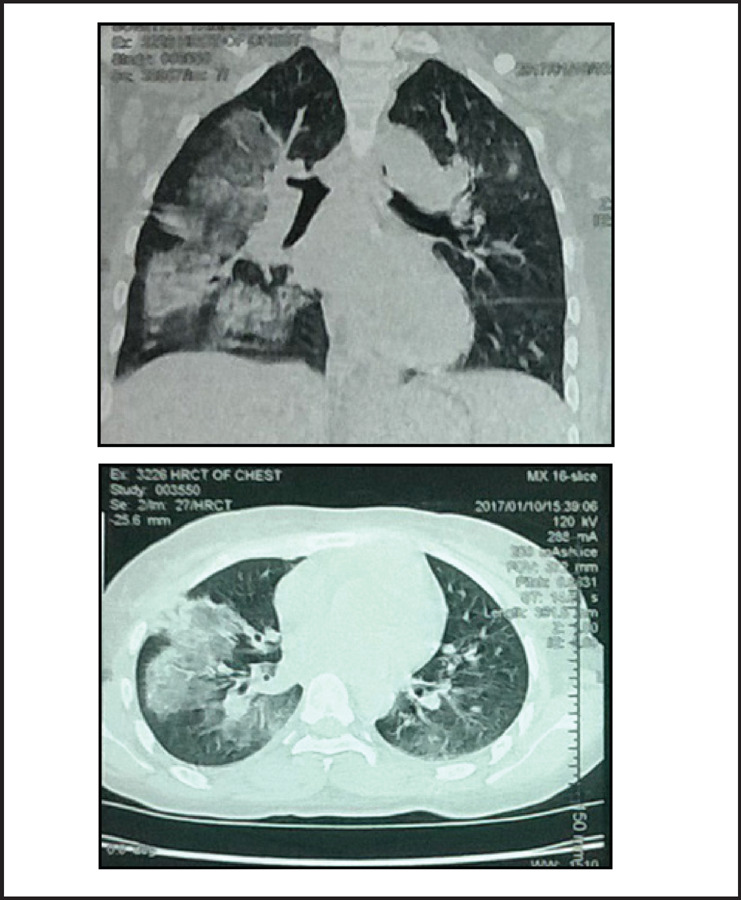
HRCT chest showing bilateral areas of consolidation and ground glass opacities, more marked on the right side.

A 22-year old lady presented with dyspnoea on exertion and cough with hemoptysis of two weeks duration. Dyspnoea was present on minimal exertion and she coughed out fresh bright red blood. After a week of these symptoms she developed oliguria and bilateral leg swelling. However, she did not complain of hematuria, fever, joint pain, skin rash or weight loss. On examination, she had pallor and bilateral pitting pedal edema. Respiratory rate was 28/min, pulse rate was 88/min and blood pressure was 138/86 mmHg at presentation. Chest examination revealed presence of bilateral basal crepitations, more prominent on the right side. Skin, cardiovascular, abdomen and neurological examinations were unremarkable. Her hemoglobin was 8.6 gm/dL, TLC was 11800/cumm and platelet count was 2,24,000/cumm. Urine examination revealed 2+ proteinuria with 6–8 RBC's/hpf and 24-hour urine protein was 2.31gms. Blood urea was 64 mg/dL and serum creatinine was 1.7 mg/dL. Serum albumin was 3.1 gm/dL, otherwise the liver function tests were normal. Chest X-ray revealed presence of bilateral fluffy alveolar opacities in the middle and lower zones (Figure 1). HRCT revealed patchy areas of consolidation and ground glass opacities in bilateral lung fields, which were more prominent in the lower lobes and on the right side (Figure 2). ANA titres were raised showing a homogenous pattern and anti dsDNA was positive. Serum complement levels were low (22 mg/dL). Anti GBM antibody, MPO and PR3 ANCA tests were negative.

**Figure 3.**
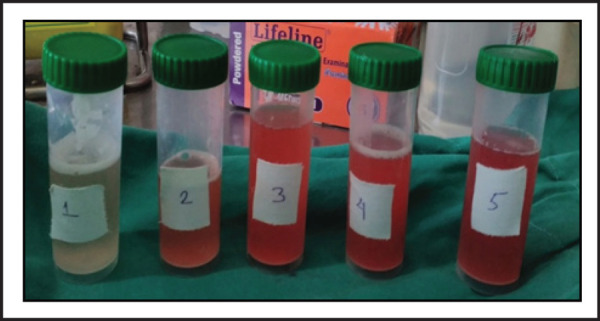
Bronchoalveolar lavage fluid showing aliquots with increasing hemorrhagic returns.

**Figure 4.**
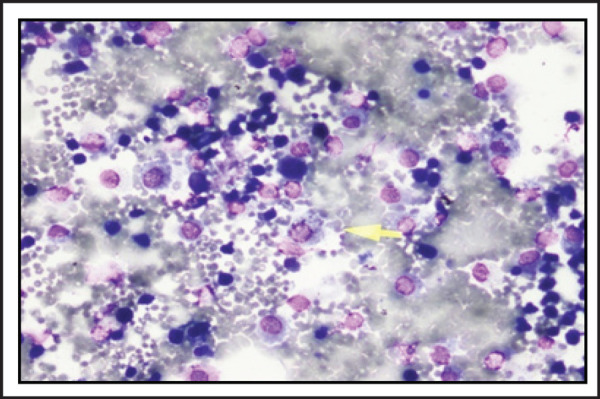
BAL fluid cytology showing plenty of hemosiderin laden macrophages.

With a suspicion of diffuse alveolar hemorrhage in the setting of SLE, a bronchoscopy was performed. Bronchoalveolar lavage (BAL) was done by instilling aliquots of 20–40 ml saline into the right lower lobe segments and collecting the return in separate containers. Serial BAL aliquots showed progressive increase in the haemorhagic return (Figure 3). Cytology revealed presence of plenty of alveolar macrophages which were laden with haemosiderin on iron stain. (Figure 4). BAL fluid AFB stain, bacterial cultures and fungal cultures were negative. Since she had renal involvement, a kidney biopsy was also done, which revealed focal segmental necrotizing vasculitis with presence of granular immune deposits of IgG, IgM, C3 and C1q. Based on the clinical, radiology, BALF, serology and histological findings a final diagnosis of SLE with Class III A/C lupus nephritis and diffuse alveolar hemorrhage was made.

She was initiated on treatment with intravenous pulse Methylprednisolone for three days and plasma exchange on alternate days. Oral Cyclophosphamide was started at a dose of 2mg/kg after explaning the adverse effects including risk of ovarian failure and infertility. After six sessions of plasma exchange there was significant clinical resolution of dyspnea, hemoptysis and the kidney functions normalized. The patient was discharged on day 15 on oral Prednisolone and Cyclophosphamide.

## DISCUSSION

Diffuse alveolar hemorrhage is a life threatening complication of systemic vasculitis that has high mortality.^5^ Cardinal features of DAH are hemoptysis, anemia, diffuse pulmonary infiltrates and hypoxemic respiratory failure.^1,2^ Hemoptysis may be absent in one third of patients because of the large alveolar volume.^6^ Fever and chest pain are uncommon symptoms. Symptoms of underlying vasculitis or collagen vascular disorder may accompany them. In the index patient, the cardinal symptoms of DAH were present at the onset of illness. She also had features of acute nephritis as she had pedal edema, proteinuria, active urinary sediments, raised serum creatinine levels. DAH should always be suspected in patients who have compatible clinical symptoms and predisposing conditions. Differential diagnoses include acute pulmonary edema, opportunistic infections and primary pulmonary manifestation of underlying collagen vascular disease. These need to be excluded before the treatment is initiated.

Bronchoscopy and BAL is an extremely useful investigation as it can document the presence of alveolar hemorrhage and also exclude infections. In hemorrhages that originates from the airways, sequential lavage leads to progressive decrease in hemorrhagic return. On the contrary, if the hemorrhage originates in the alveoli, the subsequent aliquots of BAL are more hemorrhagic as the return from the distal and number of alveoli increases.^7^ An increasing hemorrhagic return of the BAL fluid in subsequent aliquots during bronchoscpy clinched the diagnosis of alveolar hemorrhage in our case. The RBC's in the alveoli are engulfed by the resident alveolar macrophage which stain positive for hemosiderin on iron stain. Detection of hemosiderin laden macrophage has a high sensitivity and specificity in the diagnosis of DAH.^8^

DAH is a catastrophic complication in SLE with a reported frequency of 2–5% and mortality as high as 50%.^9,10^ DAH occurs as initial manifestation of SLE in 20% cases.^11–14^ It is almost invariably accompanied with severe nephritis, as occurred in the index case. BAL is mandatory to rule out lupus pneumonitis and secondary infection.

Since DAH has a high mortality, immediate treatment with immunosuppressive agents is imperative once the diagnosis is established. The standard treatment is a combination of pulse intravenous methylprednisolone for three days and cyclophosphamide. Rituximab, a monoclonal antibody against CD20 positive B lymphocytes, is used in refractory lupus and unlike cyclophosphamide does not cause ovarian failure or infertility. However, its role as a first line therapy is not well established in randomized trials to date.^15^ Plasma exchange is useful in clearing the immune complexes from the circulation and hence the immune mediated injury. Plasma exchange has been used successfully as adjunctive treatment of DAH in lupus.^16,17^

DAH in systemic lupus erythematosus is rare but life-threatening, hence requires a high index of suspicion, early diagnosis by bronchoalveolar lavage and prompt treatment with systemic steroid and immunosuppressant to reduce morbidity and mortality.

## Consent

JNMA Case Consent Form was signed by the patient and the original article is attached with the patient's chart.

## Conflict of Interest

**None.**
